# Strategic Grassland Bird Conservation throughout the Annual Cycle: Linking Policy Alternatives, Landowner Decisions, and Biological Population Outcomes

**DOI:** 10.1371/journal.pone.0142525

**Published:** 2015-11-16

**Authors:** Ryan G. Drum, Christine A. Ribic, Katie Koch, Eric Lonsdorf, Evan Grant, Marissa Ahlering, Laurel Barnhill, Thomas Dailey, Socheata Lor, Connie Mueller, David C. Pavlacky, Catherine Rideout, David Sample

**Affiliations:** 1 U.S. Fish and Wildlife Service, Bloomington, MN, United States of America; 2 U.S. Geological Survey, Wisconsin Cooperative Wildlife Research Unit, Madison, WI, United States of America; 3 U.S. Fish and Wildlife Service, Division of Migratory Birds, Marquette, MI, United States of America; 4 Franklin and Marshal College, Lancaster, PA, United States of America; 5 U.S. Geological Survey, Patuxent Wildlife Research Center, SO Conte Anadromous Fish Laboratory, Turners Falls, MA, United States of America; 6 The Nature Conservancy, Brookings, SD, United States of America; 7 U.S. Fish and Wildlife Service, Inventory and Monitoring, Athens, GA, United States of America; 8 National Bobwhite Conservation Initiative, Columbia, MO, United States of America; 9 U.S. Fish and Wildlife Service, Anchorage, AK, United States of America; 10 U.S. Fish and Wildlife Service, Waubay Wetland Management District, Waubay, SD, United States of America; 11 Bird Conservancy of the Rockies (formerly Rocky Mountain Bird Observatory), Brighton, CO, United States of America; 12 U.S. Fish and Wildlife Service, East Gulf Coastal Plain Joint Venture, Auburn University, AL, United States of America; 13 Wisconsin Department of Natural Resources, Madison, WI, United States of America; U.S. Geological Survey, UNITED STATES

## Abstract

Grassland bird habitat has declined substantially in the United States. Remaining grasslands are increasingly fragmented, mostly privately owned, and vary greatly in terms of habitat quality and protection status. A coordinated strategic response for grassland bird conservation is difficult, largely due to the scope and complexity of the problem, further compounded by biological, sociological, and economic uncertainties. We describe the results from a collaborative Structured Decision Making (SDM) workshop focused on linking social and economic drivers of landscape change to grassland bird population outcomes. We identified and evaluated alternative strategies for grassland bird conservation using a series of rapid prototype models. We modeled change in grassland and agriculture cover in hypothetical landscapes resulting from different landowner decisions in response to alternative socio-economic conservation policy decisions. Resulting changes in land cover at all three stages of the annual cycle (breeding, wintering, and migration) were used to estimate changes in grassland bird populations. Our results suggest that successful grassland bird conservation may depend upon linkages with ecosystem services on working agricultural lands and grassland-based marketing campaigns to engage the public. With further development, spatial models that link landowner decisions with biological outcomes can be essential tools for making conservation policy decisions. A coordinated non-traditional partnership will likely be necessary to clearly understand and systematically respond to the many conservation challenges facing grassland birds.

## Introduction

### 1.1 Background: The “Problem” of Grassland Bird Conservation

Grassland birds are among the fastest and most consistently declining birds in North America [1, 2]. The widespread loss of grasslands and subsequent degradation of remaining grassland habitat throughout the breeding grounds has been well-documented and is generally accepted as a major driver of grassland bird population declines [3–6]. In the United States, 48% of grassland bird species are considered to be of conservation concern and 55% are exhibiting significant population declines [7]. With historically low population numbers and continued declines in the availability and quality of habitat throughout each phase of their annual cycles, the prospects for many grassland bird species are not positive (e.g., [4, 8]).

Grassland losses throughout the breeding grounds have been rapid and geographically extensive, most notably in the agricultural landscapes of the Midwest and eastern Great Plains, where grasslands had historically encompassed some of the best agricultural soils on Earth. Most native tallgrass prairie had disappeared from the Midwest by the dawn of the 20^th^ century [9]. Native grasslands, particularly the tallgrass prairie, are now viewed as one of the most “endangered” ecosystems in the world [5, 10]. Recent trends suggest that the rate of grassland losses in the U.S. corn belt region are comparable to deforestation rates in Brazil, Malaysia, and Indonesia—grassland loss rates that have not been observed since the rapid industrialized agricultural expansion period of the 1920s and 1930s [11]. Approximately 23 million acres of grassland, wetland, and shrubland in the US were converted to row crop production between 2008 and 2011 [12], comparable to the area encompassed by the state of Indiana; approximately 10 million acres of USDA Conservation Reserve Program (CRP) easements were converted to cropland between 2007 and 2013 [13]. The continued intensification of farming practices, conversion of grasslands into annually tilled crops, forest succession, urbanization and energy development have further compounded the degradation of the largely-fragmented grassland habitat that remains [14–16].

Grassland birds face many challenges in addition to the direct loss of habitat on the breeding grounds. Most grassland birds are migratory. They nest and produce young in the grasslands of the United States and Canada and then fly south to winter in the southern US, Caribbean, or Central and South America before returning for another breeding season. Ecology on the breeding grounds, while more easily studied and thus generally better understood, tells an important but incomplete story. Information on the wintering grounds is more limited [17], although recent work suggests that many of the same underlying drivers of habitat conversion and degradation on the breeding grounds likewise impact the wintering grounds [18–20]. Much uncertainty remains with respect to the factors limiting grassland bird populations during migration and wintering periods. This is an area of growing research. Conservation work linking the full annual cycle has been applied to some Neotropical migratory birds (e.g., [21]), but this avenue of research is largely in its infancy for grassland birds. Incorporating the often-unknown spatial and temporal complexities of grassland bird annual cycles remains a significant problem for conservationists attempting to understand how to guide policies and leverage limited resources strategically to best manage for these birds.

Long-term success for grassland bird conservation will likely depend on effective allocation strategies focused on private lands. The extent to which grassland bird conservation is reliant on influencing private landowner decisions poses a unique challenge. Remaining grasslands in the U.S. are approximately 85% privately owned, totaling about 300 million acres, with approximately 82% of current grassland bird populations distributed directly on private lands [8]. Devising conservation programs for private lands requires an understanding of how socio-economic factors influence decision-making processes of landowners [22].

Federal laws exist to protect migratory birds. The Migratory Bird Treaty Act (MBTA) of 1918 declared that all migratory birds are protected by law, and the US Fish and Wildlife Service (USFWS) is charged with Federal Trust responsibility for these species. However, population declines caused by habitat conversion or management practices on agricultural lands do not typically fall under the scope of the MBTA [23, 24]; only those species that fall under the jurisdiction of the Endangered Species Act have protection from indirect “take” due to habitat loss. Executive Order 13186 mandates that the USFWS coordinate, develop, and implement bird conservation activities with other Federal agencies. Further, many state wildlife agencies and non-governmental organizations (NGOs) are charged with protecting and enhancing natural resources in concert with multiple public uses on their respective lands (e.g., Fish and Wildlife Conservation Act of 1980). State agencies also manage for a variety of resident game species that depend on grassland habitats. While it is commonly acknowledged that private lands are important for grassland bird conservation [5, 17], there have been few attempts to investigate the complex linkages between policy drivers, cumulative actions of private landowners, and the resulting grassland bird population outcomes [25].

Many partners have been working for years on grassland bird conservation issues, including Migratory Bird Joint Ventures, USFWS, various other federal and state agencies, NGOs, and academic institutions. However, an effective framework for integrating and coordinating strategic grassland bird conservation has remained elusive, in large part because the information and decisions necessary to be successful in this arena are beyond the scope and responsibility of any single entity or academic discipline [4].

Structured Decision Making (SDM) provides a useful framework for increasing transparency and defensibility in complex, multi-stakeholder decision making while also exploring tradeoffs of alternative strategies and helping to identify important information gaps [26, 27]. We utilized the SDM process to 1) identify where and how to conserve grassland bird populations in a way that integrates actions on the breeding, migration, and wintering grounds, 2) better understand the variety of perceived problems associated with grassland bird conservation across the annual cycle, and 3) develop a portfolio of innovative solutions that demonstrate how a system of integrated social-biological spatial models can inform a strategic response for what many perceive to be a “wicked” problem [28]. Results from this process include a framework for strategic grassland bird conservation, a rapid prototype spatial modeling approach intended to serve as proof of concept for linking policy alternatives with grassland bird population outcomes, and a series of insights to guide grassland bird conservation partners.

## Methods

### 2.1 Workshop details

We convened a group of experts working on different aspects of grassland bird conservation for a one-week SDM workshop in 2011. Participants were selected to cover a broad range of scientific, management, and geographic expertise. Individuals selected were working on grassland bird conservation issues east of the Rocky Mountains and were chosen with the intent of balancing the following areas of expertise: federal and state migratory bird biologists, regional conservation planners, biometricians, researchers, non-governmental conservation organizations, and local area managers. Our goals for the workshop were to 1) clearly articulate the perceived problem(s) limiting successful grassland bird conservation, 2) identify strategic opportunities and innovative solutions to promote more effective grassland bird conservation, and 3) develop a framework to orient various partners with different missions and skillsets into a value-added holistic system.

#### SDM process and scope

Briefly summarized, the SDM process begins with a focused evaluation of the problem of interest. The “problem” is then addressed in terms of a fundamental objective and sub-objectives, including measurable attributes that describe each level of objectives. Alternatives are identified and framed in terms of measurable outcomes that directly address the defined objectives. Models are then developed and applied to evaluate consequences for each alternative. Tradeoffs are then considered in the context of the identified objective(s) [29].

We focused on grassland birds that breed east of the Rocky Mountains (including Canada) and which migrate and/or over-winter in Mexico, South and Central America. The problems facing grassland birds extend beyond this geographic area for some species. However, participants agreed that the chosen geography encompassed the majority of grassland birds in North America and was appropriate given the expertise of participants.

#### Problem framing

A substantial portion of the workshop was focused on eliciting the problem context. The problem was described as the observed ongoing declines of most migratory grassland bird species’ populations, confounded by pervasive uncertainty regarding cause-effect relationships throughout the annual cycle and a general lack of clarity about how to coordinate a viable systematic (i.e., socio-economic-biological) response that would maximize grassland bird population outcomes. Participants emphasized that conservation work for grassland birds entails many scientific uncertainties, economic constraints, and complex social-political and economic drivers of landscape change at various scales.

Effectively responding to these challenges will likely require a systemic understanding of grassland bird natural histories, broad-scale migration patterns, private landowner decision making, and the social-economic “landscapes” within which these species are integrated throughout their annual cycle of breeding, migration, and wintering periods—which can vary widely among the different grassland bird species. Important decisions affecting grassland birds are made at various inter-related scales, a combination of national and international socio-economic policy decisions, agency and organizational resource allocation decisions, conservation priorities, and ultimately individual private landowner decisions on the ground.

Workshop participants worked to develop a conceptual framework that describes the complex components of this problem and then used the resulting framework to demonstrate how an integrated modeling process based on private landowner decisions can be used to elucidate alternative solutions and explore their respective tradeoffs. While these results focus primarily on policy decisions, the framework itself is designed to also inform decisions related to research priorities, model development, and coordination needs.

### 2.2 Modeling Framework Overview

Workshop participants developed a series of linked agent-based landowner decision and spatial biological models to represent the coupled human-environmental system that drives grassland bird populations throughout their annual cycle. Models were designed to be simple to illustrate how the proposed framework can be applied to evaluate alternative land use decisions, fully acknowledging that species-specific data and models, including more robust social science information, could be incorporated to strengthen this approach (see S1 and S2 Appendices for model details, limitations and critical assumptions). This could be particularly effective if implemented in an Adaptive Management context [30, 31].

#### The fundamental objective

Based on the current status and population trends of the majority of migratory grassland bird species, our fundamental objective was:

Sustain and restore migratory grassland bird populations that breed east of the Rocky Mountains (including Canada).

Participants did not delve into a species-by-species population target-setting exercise, but rather specified that the appropriate metric for evaluating success should be based on biological outcomes (i.e., grassland bird population trends, in this case for a hypothetical population). While current population estimates are available for many grassland bird species [32], the group decided species-specific applications would be better explored through existing partnerships. We decided to focus generally on grassland birds collectively, to use hypothetical species to demonstrate concepts.

Additionally, given the importance of human activities in providing habitat for grassland birds, and the fact that the majority of restorable grasslands are privately owned, we identified two means objectives, each believed to directly support the fundamental objective.

*Means Objective 1*: *Protect and restore as much grassland habitat as possible—*The most direct way to produce more birds is to provide more habitat. Therefore, we proposed that the primary means for increasing the population of grassland birds was through the protection and restoration of habitat, primarily on the breeding grounds but also encompassing wintering and migration habitats.

*Means Objective 2*: *Maximize the likelihood that private landowners will choose to provide habitat for grassland birds—*Workshop participants recognized the importance of private landowner decisions in determining how much and where grasslands remain or could be restored. With the predominance of land in private ownership, restoring land to grassland cover will depend on the willingness of private landowners to sell their land to conservation organizations, practice “bird-friendly” grass-based farming, or participate in conservation programs (e.g., the Conservation Reserve Program). In doing so, private landowner participation effectively translates into more habitat, which ultimately produces more birds.

In this case, one could view the primary “decision maker” as being high-level policy makers (e.g., legislators, senior administrators, etc.) whom might consider various policy alternatives and presumably would decide to enact one or more of them. However, given that few policy makers focus on grassland birds as a primary objective, it is likely more meaningful to consider this framework as a multi-faceted approach to guide a combination of research, modeling, monitoring, advocacy, and coordination of priorities for all partners focused on grassland bird conservation. High-level policy decisions will be an important component of the solution; however, workshop participants continually emphasized the need for a systematic approach to align partners in such a way that spans well beyond any single decision-making sector.

### 2.3 Conceptual Framework and Policy Alternatives

**Fig 1:**
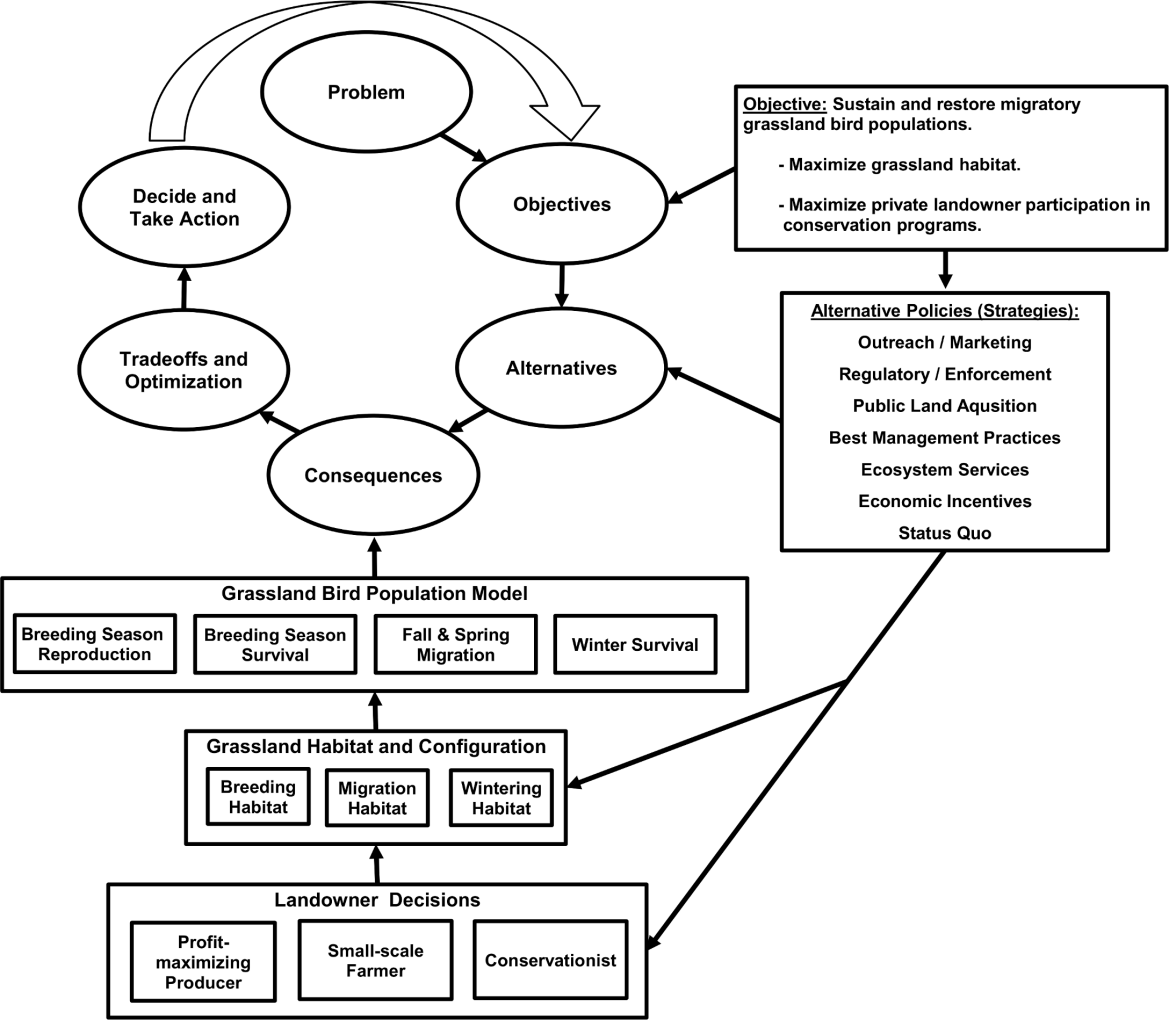
Integrated Modeling Framework. Policies influence landowner decision-making, the effect of which depends on the different landowner types’ respective value systems. Landowner decisions affect habitat quantity and configuration, which function as inputs for the Grassland Bird Population Model.

We developed a conceptual framework for linking policy alternatives, landowner-decisions, habitat configuration, and spatial biological models, to evaluate population outcomes for grassland birds (Fig 1; see S1 and S2 Appendices for complete modeling details). The foundational assumption underlying the framework was that grassland bird populations were driven by the amount and configuration of grassland habitat (during breeding, migration, and wintering periods), which are in turn determined largely by private landowner decisions. We assumed that publicly owned grasslands are permanently protected and thus can effectively be held as a constant. Therefore, we exclusively explored the implications of alternatives impacting private lands.

This framework consists of two key components represented by two linked models, an agent-based “Landowner Model” (S1 Appendix) and a spatially explicit full-annual-cycle “Grassland Bird Population Model” (S2 Appendix) (Fig 1). The land cover output of the Landowner Model—determined by landowner types and their respective value systems, adjusted in response to policy alternatives—serves as the land cover input for the Grassland Bird Population Model.

#### Policy alternatives

We identified a series of policy options related to grassland bird conservation, modeled their respective influence on private landowners and then estimated outcomes for grassland birds. This approach was designed to identify and compare strategic opportunities and to provide insight regarding which strategies are worth further consideration; at which point assumptions would need to be further evaluated and more detailed statistical models could be developed and integrated. We analyzed policies individually for simplicity. Participants assumed that, in reality, leveraging multiple policies would likely be more effective, whenever possible. We attempted to identify a reasonably diverse and comprehensive set of policy alternatives though this list is by no means exhaustive.

Alternative policies were grouped into seven categories, which were applied to the three landowner types’ value systems:

1. **Outreach / Marketing:** Foster the culture of grassland preservation and restoration. For instance, we might approach high-profile celebrities to publicly champion grassland conservation via social media. Additional ideas included developing an “Adopt a Prairie” program, collaborating with the America’s Great Outdoors campaign, promoting ecotourism, collaborating with the Wildlife Conservation Society to use bison as a means to promote grasslands (akin to Smokey the Bear), and engage and incentivize landowners to collaborate with private entities, such as the National Wildlife Refuge System Friends group, Cattlemen’s Associations, and other NGOs to champion policy development.
2. **Regulatory / Enforcement:** Improve regulatory and enforcement policies both domestically and internationally (e.g., a “no net loss” policy for grasslands), with an increased emphasis on countries where grassland birds migrate through or overwinter (e.g., new policies addressing specific threats such as pesticide use, improved enforcement of existing government regulations).
3. **Public Land Acquisition:** Promote existing grassland habitats in public ownership and new public ownership (e.g., improve integration of wetland restoration and flood mitigation projects with grassland habitat conservation, advocate for increased funding for acquisition of public grasslands, and designate additional grassland National Parks (i.e., as the “American Serengeti”)).
4. **Best Management Practices (BMP):** Use policy tools, based on empirical data, to promote the widespread implementation of best management practices with high likelihoods of success for meeting grassland birds’ life history needs within working agricultural landscapes.
5. **Ecosystem Services Payments:** Develop markets and/or provide tax-based subsidies for landowners maintaining grasslands for the ecosystem services they provide (e.g., direct payments or tax credits for pollinator services, sequestering carbon, hunting opportunities, water quality benefits, protecting view sheds).
6. **Economic Incentives:** Design policies to support more competitive incentive programs for landowners alongside market-based solutions. Actions described under this alternative, largely related to U.S. Farm Bill Policy, included: incorporating flexibility to change rental rates in response to changes in commodity prices, allowing for state and regional flexibility in setting rental rates, reducing or eliminating federal ethanol subsidies, and focusing on creative alternatives to make energy development more grassland friendly (e.g., cellulosic ethanol production). Market-based solutions could also be encouraged through certification and trade infrastructure for grassland bird-friendly beef and other related products.
7. **Status quo** (the No-Action Alternative): No additional focus on improving the protection or management of grassland birds or habitats anywhere in the annual cycle.

### 2.4 Evaluating Consequences

#### Landowner decision model: an agent-based land cover choice model

We developed a simple agent-based model (ABM) of land cover change (see S1 Appendix for ABM model details). ABMs of land cover change are used to determine how local level decision-making leads to land use and land cover change [33]. In our simple prototype, a landowner chose one of three options for land use: agriculture, grassland, or forest. In using the ABM, we expected a rational decision to be made that maximizes utility for a landowner but which differed because there were several types of landowners, each with different value systems (e.g., [34]). We applied this framework to evaluate how seven different policies might affect each of three landowner’s land cover choices. Landowner decisions, to maintain or change land cover types on their land, were modeled on a per-pixel basis.

We hypothesized three general categories of private landowners: 1) a profit-maximizing producer, 2) small-scale farmer, and 3) a conservationist. Each landowner class entailed a specific value system related to 5 specific objectives: grassland birds, carbon storage, water quality, net revenue, and biodiversity. We assumed that each landowner would attempt to maximize his/her land’s utility [35]. The importance weight attributed to each objective depended on the landowner. We assumed that: 1) the profit-maximizing producer’s decisions were dominated by expected net revenue utility, 2) the conservationist based decisions on expected utility for biodiversity and grassland birds over other metrics, and 3) the conservation-minded small-scale farmer was essentially a hybrid of these two extremes. We modeled the value system of the three landowner types, including an initial (“status quo”) value system attributed to each landowner type, and their respective responses to the various policy alternatives (Table 1).

**Table 1:** Preference weights for each landowner type, for each objective and policy. Weights were used to evaluate land cover choices for each landowner and were based on the opinions of workshop participants. Future applications of this approach could incorporate empirical social science data to inform the value weightings of an agent-based model.

**Profit-maximizing Producer**							
	Outreach/Marketing	Regulatory/ Enforcement	Public Lands	Best Management Practices	Ecosystem Services Payments	Economic Incentives	Status Quo
Birds	0.15	0.00	0.00	0.00	0.03	0.03	0.00
Carbon	0.21	0.08	0.00	0.00	0.15	0.13	0.00
Water Quality	0.21	0.08	0.00	0.00	0.15	0.13	0.00
Financial Profit	0.30	0.83	1.00	1.00	0.51	0.67	1.00
Biodiversity	0.12	0.00	0.00	0.00	0.15	0.03	0.00
**Small-scale Farmer**							
	Outreach/Marketing	Regulatory/ Enforcement	Public Lands	Best Management Practices	Ecosystem Services Payments	Economic Incentives	Status Quo
Birds	0.23	0.20	0.20	0.23	0.24	0.26	0.23
Carbon	0.07	0.00	0.00	0.06	0.00	0.00	0.00
Water Quality	0.10	0.10	0.20	0.16	0.17	0.15	0.09
Financial Profit	0.33	0.50	0.40	0.32	0.34	0.37	0.45
Biodiversity	0.27	0.20	0.20	0.23	0.24	0.22	0.23
**Conservationist**							
	Outreach/Marketing	Regulatory/ Enforcement	Public Lands	Best Management Practices	Ecosystem Services Payments	Economic Incentives	Status Quo
Birds	0.24	0.29	0.29	0.25	0.26	0.27	0.30
Carbon	0.17	0.11	0.12	0.15	0.10	0.11	0.12
Water Quality	0.17	0.11	0.12	0.18	0.18	0.16	0.12
Financial Profit	0.19	0.23	0.24	0.20	0.23	0.24	0.24
Biodiversity	0.24	0.26	0.24	0.23	0.23	0.22	0.21

Social science information could help to better inform the classification, abundance, and spatial distribution of different landowner types and their respective value systems. The simplified treatment of these values systems in our model is intended to serve as a temporary placeholder—a beacon for future social science research—until more precise estimates are available.

#### Integrating the land cover choice and grassland bird models

We generated five 1.44-km^2^ “landscapes” (grids) of 1600, 30-meter pixels. We initially randomly assigned each pixel as grassland, agriculture or forest with the probability of each choice being determined by the alternative-generated results of the ABM. For this rapid prototype effort, we assigned one landscape to represent the breeding area, three to be migratory stopover areas, and one to be the wintering ground. The landscapes that were generated by this process then served as the inputs for a series of linked spatially explicit biological models designed to estimate grassland bird population outcomes in response to land cover change.

#### Grassland bird population model

We developed a prototype spatially explicit annual cycle population model for grassland birds (see S2 Appendix for grassland bird population model details). Like many birds, the majority of grassland birds have four phases of their annual cycle that roughly correspond to the four seasons but occur in different geographic locations, including: a summer breeding season in the north, a wintering period in the south and two migratory periods in the spring and fall when they travel between wintering and breeding areas. Thus, we assumed that reproduction occurs in one season and location and the majority of mortality occurs during the other three seasons. To reflect this basic biology, we modeled the population of birds in the breeding area at the start of the breeding season during year t (N_t_) as:

$$N_{t}=N_{t\text{-}1}(1+R_{b})S_{f}S_{w}S_{s}$$

where *R*_*b*_ was the reproductive output during the summer breeding season and *S*_*f*_, *S*_*w*_, and *S*_*s*_ were the survival probabilities during fall migration, over-winter, and spring migration, respectively. We defined the values of each of these steps as a function of a landscape that consists of forest, grassland and agricultural patches.

Carrying capacity limits were incorporated into the grassland bird model. The amount of habitat in each of the annual cycle phases/landscapes determined the number of birds that could survive and reproduce. We used the group’s knowledge of grassland birds to determine how reproduction may change with respect to the composition and configuration of the landscape and used this information to parameterize the model described below. We determined the number of birds fledged by calculating the potential for nests to occur within a particular pixel, the survivorship probability of an individual from within an egg to independence, and the expected density of nests on the pixel.

We developed a basic representation of stopover ecology by dividing stopover survivorship of an individual pixel into two components, occupancy and survivorship. Like reproduction, each component depended on the landscape composition at different scales. Occupancy, the joint probability that a pixel is both located and utilized by migratory birds, was determined by the land cover type of the individual pixel and by the immediate surroundings. For fall and spring migration, survivorship was determined by the forage quality within a one hectare “patch”.

We represented a grassland bird’s interaction with the landscape during winter in a similar way to the migration model. However, it was simplified in that only individual pixel-level determined survivorship (i.e., no neighborhood effect was incorporated). Results were summarized for a 30-year time period.

## Model Results

### Integrated modeling framework

We demonstrated the utility of an integrated social-biological modeling framework (Fig 1) by exploring the implications of various policy alternatives. The resulting modeling framework portrays linkages between landowner value systems, land use and land cover change, and full annual-cycle grassland bird population outcomes. In addition to informing policy priorities, the framework highlighted important opportunities for innovative statistical modeling efforts and also helps to inform potential research priorities that could help to parameterize related models. Modeling results were hypothetical, based on expert opinion, yet were nonetheless informative; this work provides insight into strategic policy options that could maximize grassland bird population outcomes.

### Grassland Bird Population Growth under Different Policy Alternatives (Evaluation of Trade-offs)

**Fig 2:**
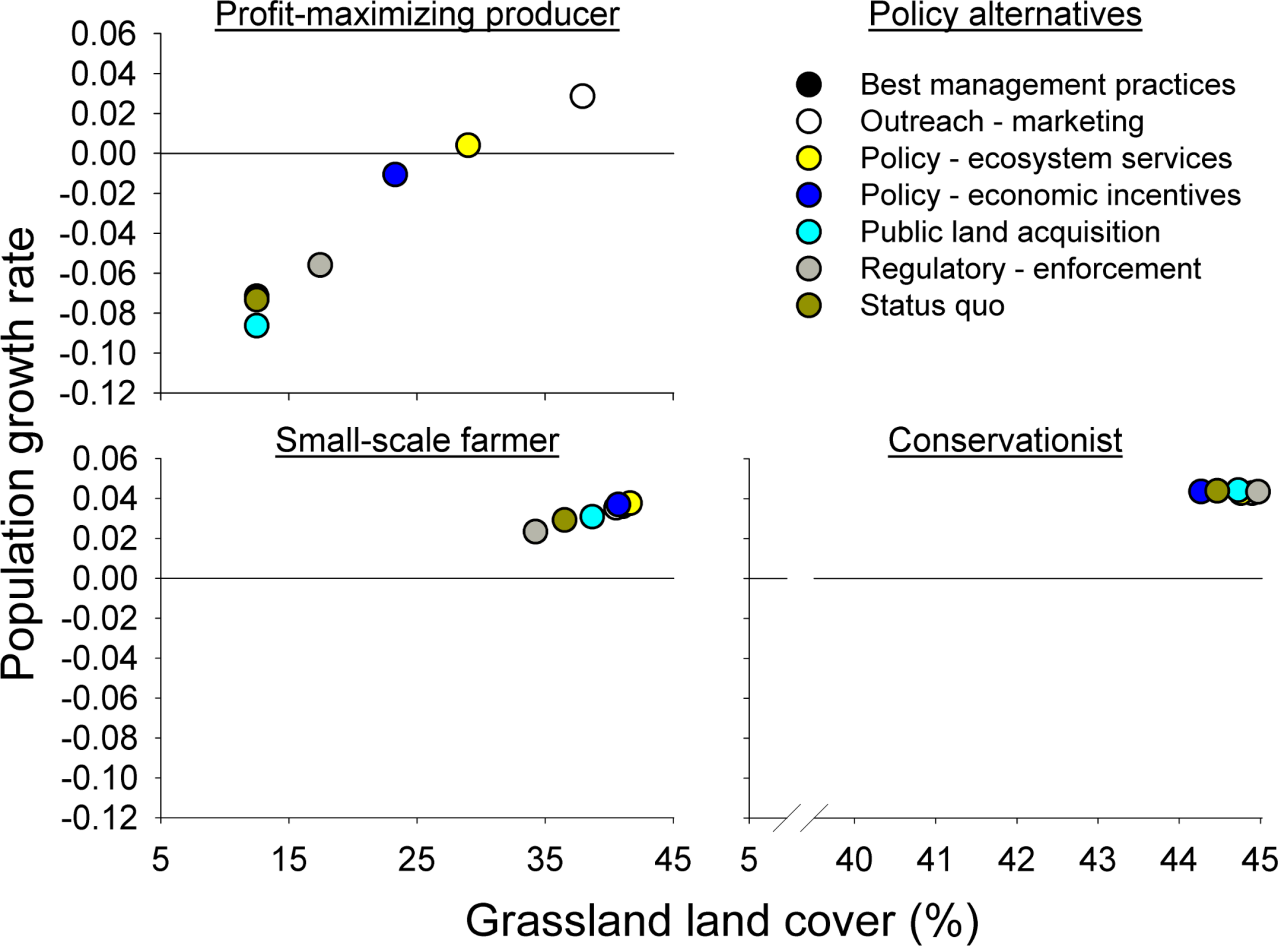
Resulting hypothetical grassland bird population growth rates over a 30-year time period, in response to various policy alternatives. Results indicate different potential impacts of policies, varying by landowner types, in relation to the percentage of grassland cover in the landscape.

Grassland bird population outcomes were estimated for each landowner-policy combination (Table 2); outcomes varied according to policy alternative and landowner type. Most alternatives associated with profit-maximizing producers’ landscapes resulted in negative grassland bird population growth rates; positive growth rates were associated with small-scale farmers’ and conservationists’ landscapes (Table 2, Fig 1 and Fig 2). Model results indicated that the greatest potential for positive change to grassland bird population levels appeared to depend on influencing the decisions made by profit-maximizing producers, suggesting that some form of direct economic incentives will likely play an important role in driving grassland bird population outcomes. The two policy alternatives that resulted in positive grassland bird population growth rates within profit-maximizing producers’ landscapes were Outreach/Marketing and Ecosystem Services Payments.

**Table 2:** The effect of each policy and landowner on the expected growth of grassland bird populations where alternatives and values were determined by workshop participant expert opinion. Values indicate average population growth rates, per year, after a 30-year simulation period, in response to the respective policy alternatives, for each landowner type.

Policy Alternative	Simulated Grassland Bird Annual Population Growth Rates		
	Profit-maximizing Producer	Small-scale Farmer	Conservationist
**Outreach / Marketing**	0.0287	0.0353	0.0427
**Regulatory / Enforcement**	-0.0569	0.0235	0.0431
**Public Land Acquisition**	-0.0863	0.0308	0.0442
**Best Management Practices**	-0.0715	0.0361	0.0436
**Ecosystem Services**	0.0041	0.0376	0.0427
**Economic Incentives**	-0.0106	0.0370	0.0435
**Status Quo**	-0.0733	0.0292	0.0438

## Discussion

Grassland bird habitat has declined substantially and continues to do so [7, 22]. Conservation efforts may need to be strategically targeted, leveraged across sectors to produce habitat sufficient to maintain and, in many cases, restore populations. This problem is particularly complex because grassland bird population outcomes depend largely on decisions made by many individual private landowners, dispersed throughout the geographies associated with the full annual cycle of these migratory birds.

We developed a rapid prototype model that relates landowner decisions to changes in grassland habitat throughout the annual cycle, which enabled us to explore how different policies might impact grassland bird populations. This modeling framework is novel in that it formally integrates the modeling of grassland bird populations throughout the annual cycle with private land-owner value systems. We recognized that detailed information is not immediately available for all annual cycle stages (especially during migration) or private landowner value systems. However, a lack of data should not justify inaction; rather, as we have demonstrated, it is possible to make progress through informed assumptions and use an integrated interdisciplinary modeling framework to help coordinate future research and monitoring efforts.

Strategies for grassland bird conservation can attempt to leverage opportunities that align most effectively with existing landowner value systems (e.g., providing direct economic incentives to make grassland more profitable) or perhaps work to change these value systems entirely (i.e., increase the perceived non-monetary values associated with grasslands); it could also be possible to change the proportions of different landowner types (e.g., to reduce the total number of profit-maximizing products and/or increase the number of conservationists). Our process highlighted the need to think creatively, to incorporate both biological and social science into conservation policy strategies, along with economics, to guide the focus of limited conservation funding towards projects or policies that have the highest likelihood of success.

We evaluated each landowner-policy combination individually. However, the most effective strategies are likely to entail multiple policy decisions being implemented simultaneously, affecting a dynamic and spatially diverse landscape of diverse habitats and social value systems. Future advancements on this topic could evaluate a portfolio of options that includes combinations of policy alternatives to better understand how to systematically maximize grassland bird populations.

To date, grassland bird conservation efforts have primarily focused on restoring or preserving habitat to halt grassland bird population declines [5]. In doing so, the conservation community has generally responded in a piecemeal fashion (e.g., during one season within the annual cycle, for single species or single geographies; [36, 37]), and yet many grassland bird populations continue to decline as habitat is lost in both the breeding and wintering grounds [5, 6, 19, 20]. The insights derived from our modeling framework underscore the importance of making connections in an interdisciplinary, applied, and strategic way. While our modeling effort was largely theoretical, we arrived at the following insights that can inform a strategic path forward:

1. Social value systems associated with different landowner types are important drivers of grassland bird population outcomes and can likely be influenced by national, regional, and local policies/programs. An integrated modeling framework can elucidate important leverage points and highlight the most effective strategies for positively impacting grassland bird population outcomes.
2. Grassland bird populations are embedded within a complex socio-economic-biological system. Additional coordination may be necessary to ensure adequate integration across partnerships (and academic disciplines), to promote the integration of research and models needed to understand and predict cause-effect relationships. Model integration and ultimate convergence around a coherent strategy is by no means assured.
3. Policies that incentivize activities on private land will continue to be very important for successful grassland bird conservation. The roles of small farms and conservation-minded landowners are notable, though they may be overshadowed by the collective impacts of large-scale profit-driven agricultural. Our results suggest that large-scale farmers hold the most sway in determining grassland bird population outcomes. Impacting large-scale farmers’ decisions to participate in conservation programs may be the most effective strategy to positively impact grassland bird populations; doing so will likely depend on influencing the economics underlying land use decisions.
4. Additional monitoring and research on grassland bird migration and wintering ecology is essential for ensuring that strategic investments in breeding ground conservation are not adversely affected by activities elsewhere in the annual cycle. Grassland bird breeding ecology is relatively well understood but much uncertainty surrounds the limiting factors associated with migration and overwintering portions of their annual cycle, which can vary greatly among species.
5. Social science data (e.g., value systems, social networks) will be more powerful if they can be linked to spatial data (e.g., demographic information, land values, land cover or other landscape context). Preferences, motivational drivers, economic considerations, and social networks of private landowners can inform policy strategies for conservation; they will be most useful if they can be directly or indirectly linked to spatial trends to predict specific opportunities and/or probabilities of success.

Our framework underscores the importance of strong and coordinated partnerships working across geographic and political scales. There are different models for how coordination can be achieved, and it remains unclear at this time which is best-suited for grassland bird conservation throughout the annual cycle, or if something entirely new is necessary. Regional alliances, international joint ventures, and community-based partnerships provide successful examples of communication, international collaboration, and expanded funding for conservation of species throughout the annual cycle [38]. Other efforts have followed a top-down or government-based approach (e.g., USFWS Migratory Bird Program Focal Species Strategy [39]) or are working in both directions (e.g., National Bobwhite Conservation Initiative [40]). For many of these partnerships, a clear need remains to fully engage business, industry, and non-governmental sectors to find economically and politically viable conservation solutions—especially solutions that extend beyond the breeding grounds [38]. Achieving success for conserving grassland bird populations will likely require a multitude of approaches; this may demand diverse partnerships in which different entities focus on different strategic leverage points (i.e., policy alternatives) such that the combined effort converges to achieve a holistic “tipping point”, resulting in that habitat necessary, throughout the annual cycle, to result in positive grassland bird population trends.

We offer this framework and related insights to help orient existing and future partnerships to design and build the linkages necessary for sustaining grassland birds. Upon completion of the 2011 SDM workshop, several advances have been made. For example, efforts are underway to develop a conservation business plan that integrates and directs land management, resource production, research, and monitoring activities throughout the full annual cycle for the Bobolink (*Dolichonyx oryzivorus)* [41]. In addition to Migratory Bird Joint Ventures [42], the maturation of USFWS-led Landscape Conservation Cooperatives [43] offers new regionally based partnerships that integrate multiple land use objectives, taxonomic groups, and interest groups to mutually manage a shared landscape. Still, the need is arguably as great as ever for proactive leadership and creative vision to effectively integrate all necessary change agents. Successful grassland bird conservation will likely require a multitude of strategies applied both through top-down policy decisions and innovative locally championed grassroots campaigns. Desired outcomes could be accomplished through many different mechanisms, and it is important to realize that the various strategy alternatives are not mutually exclusive. The efficiency and effectiveness of various partnerships and their respective actions will ultimately depend upon the degree to which they align with an overarching integrated and coordinated strategy.

## Supporting Information

S1 Appendix Agent-based Model Framework. Model details include the simple multi-attribute rating technique, utility of land cover type by objective, preference weights, and model simulation details.
(DOCX)

S2 Appendix Grassland Bird Population Model Framework. Model details describe reproduction, carrying capacity, survivorship during spring and fall migration, winter survivorship, model simulation details and population summaries.
(DOCX)
