# Supplementary material for: Strategic Grassland Bird Conservation throughout the Annual Cycle: Linking Policy Alternatives, Landowner Decisions, and Biological Population Outcomes
Source: PLoS One. 2015 Nov 16;10(11):e0142525. doi: 10.1371/journal.pone.0142525 (PMC4646652; doi:10.1371/journal.pone.0142525)
Supplement: S1 Appendix — Model details include the simple multi-attribute rating technique, utility of land cover type by objective, preference weights, and model simulation details. (DOCX) [file pone.0142525.s001.docx]

# S1 Appendix: Landowner Decision Model: An Agent-based, Multiple Objective, Land Cover Choice Model

Our models were designed to function as an integrated framework for evaluating how grassland bird populations would respond to policies that affect the composition of land cover. To accomplish this, we considered how private land owners might respond to such policies in terms of land cover choice. We then used this approach to link land cover change to grassland bird population growth. We used a simple agent-based model (ABM) of land cover change. ABMs of land cover change are used to determine how local level decision-making leads to land use and land cover change [1]. For our simple prototype, a hypothetical landowner chose one of three options for land cover: agriculture, grassland, or forest for each 30-meter pixel of their respective “landscape” (a 1.44-km^2^ grid consisting of 1600, 30-meter land cover pixels). We expected a rational decision to be made that maximized utility for a landowner but which differed among landowner types, each with different value systems (e.g., [2]). We assumed that each landowner would attempt to maximize his/her land’s utility based on five objectives: grassland birds, carbon storage, water quality, net revenue, and biodiversity. We used this framework to evaluate how seven different policies might affect each of three landowner’s land cover choices.

We represented the decision-makers (agents) as three types of private landowners: 1) a profit-maximizing producer, 2) a small-scale farmer, and 3) a conservationist. The relative importance of the five objectives to measuring overall utility (i.e. their value system) is what differentiates the landowner types. We recognized that these value systems likely exist as a much more complex gradient in the real world. The simplified treatment of these values systems in our model simply serves as an illustrative placeholder until more precise models can be incorporated into this framework; social science surveys and elicitations could help to better inform the classification, abundance, and spatial distribution of different landowner types and their respective value systems.

To determine how land cover choice affected the owner-specific perception of utility and thus their decisions about land cover change, we applied the simple multi-attribute rating technique (SMART [3]). Following SMART, the overall utility of a landscape (*U_jk_*) is the overall weighted average utility using the owner *j’s* objective weights under policy *k*:

$$U_{jk}=\sum_{i=1}^{5} w_{ijk}\sum_{c=1}^{3} p_{jc}u_{ic}$$

where *w_ijk_* represents the relative importance weight landowner *j* places on objective *i* under policy *k;* *p_jc_* is the proportion of landowner *j’s* land covered by cover type *c; u_ic_* is the utility value for objective *i* provided by cover type *c*. The values and weights associated with each landowner were determined through group discussion, serving as a hypothetical basis for the ABM.

*Determining utility of each cover type, c, for each objective, i (u_ic­­_*)*:* We assumed land owner *j’s* overall satisfaction of the composition of his/her landscape under policy *k*, *U_jk_*, was a function of the realization of five objectives: grassland birds, carbon storage, water quality, revenue, and biodiversity. To determine the combined utility across all objectives, we must know both how utility changes with changes in the value of each objective *i*, *u_i_*, as well as the relative preference weights for each objective, *w_i_*. The expected return can be converted into a common scale (S1 Table 1); in this case, an index value of 100 is the highest utility and 0 is the lowest. For example, as the amount of carbon storage increases, a landowner’s satisfaction (or utility value) will increase. Again, these served as placeholders based on logic and hypotheses of this group, which could be validated or updated using more detailed statistical spatial models alongside real-world data on land cover and landowner value systems.

**S1 Table 1. Utility of each cover type for each objective.** These utilities, *u_ic_*, were used to evaluate how each land cover, *c*, could produce each objective, *i,* and were hypothetical values determined by expert opinion of workshop participants.

|  | |  | Land cover (*c*) | | |
| --- | --- | --- | --- | --- | --- |
|  | |  | **Ag** | **Grassland** | **Forest** |
| Objective (*i*) | **Grassland Birds** | | 20 | 100 | 10 |
|  | **Carbon** | | 33 | 66 | 100 |
|  | **Water Quality** | | 5 | 100 | 80 |
|  | **Financial Profit** | | 100 | 25 | 75 |
|  | **Biodiversity** | | 10 | 100 | 80 |

*Determining preference weights for each objective, i, by each land owner, j, under each policy, k* (*w_ijk_*): Once we developed the standard utility scores for each cover type, we developed the relative importance of one metric compared to the other metrics where the sum of preference weights is equal to 1. For example, a conservationist would value production of grassland birds and biodiversity over revenue more than a profit-maximizer producer, who would value revenue much more highly than the biological objectives; thus, the conservationist will choose land cover that provides high utility to grassland birds and biodiversity whereas the profit maximizer’s land cover choice will provide high utility to financial profit. The conversion of individual objective values to utility is independent of the type of owner (S1 Table); in other words, we assumed that all owners would feel higher net revenue from their land is better than lower net revenue. However, different types of owners would rate the importance of revenue, relative to other metrics like grassland birds, differently. So the contribution of revenue to the ultimate decision is landowner-type-specific.

Through discussion, we hypothesized how each of the three types of private landowners would respond to policy alternatives in terms of their preference weights. For example, we believed that the profit maximizer would be forced to consider how their land influences carbon and water quality under an enforcement policy but not so under the status quo. We recognize that there are many more formal elicitation techniques to determine how these weights might change, so our goal is mainly to illustrate how the weights could be used. The table (S1 Table 2) illustrates the result of our discussions indicating the change in preference weights in response to policies for each land owner type. We assumed that the preference weights, *w*, depend on the type of landowner and their response to a policy alternative. Under a status quo, we assumed that: 1) the profit-maximizing producer’s decisions were dominated by expected net revenue utility, 2) the conservationist based decisions on expected utility for biodiversity and grassland birds over other metrics, and 3) the small-scale farmer was a hybrid of these two extremes.

**S1 Table 2. Preference weights for each landowner type, for each objective and policy.** These weights were used to evaluate land cover choices for each landowner and were based on the expert opinions of workshop participants. Future applications of this approach could incorporate empirical social science data to inform the value weightings of an agent-based model.

| **Profit-maximizing Producer** | | | | | | | |
| --- | --- | --- | --- | --- | --- | --- | --- |
|  | Outreach/Marketing | Regulatory/ Enforcement | Public Lands | BMP | Ecosystem Services | Farm Bill | Status Quo |
| Birds | 0.15 | 0.00 | 0.00 | 0.00 | 0.03 | 0.03 | 0.00 |
| Carbon | 0.21 | 0.08 | 0.00 | 0.00 | 0.15 | 0.13 | 0.00 |
| Water Quality | 0.21 | 0.08 | 0.00 | 0.00 | 0.15 | 0.13 | 0.00 |
| Financial Profit | 0.30 | 0.83 | 1.00 | 1.00 | 0.51 | 0.67 | 1.00 |
| Biodiversity | 0.12 | 0.00 | 0.00 | 0.00 | 0.15 | 0.03 | 0.00 |
|  |  |  |  |  |  |  |  |
| **Small-scale Farmer** | | | | | | | |
|  | Outreach/Marketing | Regulatory/ Enforcement | Public Lands | BMP | Ecosystem Services | Farm Bill | Status Quo |
| Birds | 0.23 | 0.20 | 0.20 | 0.23 | 0.24 | 0.26 | 0.23 |
| Carbon | 0.07 | 0.00 | 0.00 | 0.06 | 0.00 | 0.00 | 0.00 |
| Water Quality | 0.10 | 0.10 | 0.20 | 0.16 | 0.17 | 0.15 | 0.09 |
| Financial Profit | 0.33 | 0.50 | 0.40 | 0.32 | 0.34 | 0.37 | 0.45 |
| Biodiversity | 0.27 | 0.20 | 0.20 | 0.23 | 0.24 | 0.22 | 0.23 |
|  |  |  |  |  |  |  |  |
| **Conservationist** | | | | | | | |
|  | Outreach/Marketing | Regulatory/ Enforcement | Public Lands | BMP | Ecosystem Services | Farm Bill | Status Quo |
| Birds | 0.24 | 0.29 | 0.29 | 0.25 | 0.26 | 0.27 | 0.30 |
| Carbon | 0.17 | 0.11 | 0.12 | 0.15 | 0.10 | 0.11 | 0.12 |
| Water Quality | 0.17 | 0.11 | 0.12 | 0.18 | 0.18 | 0.16 | 0.12 |
| Financial Profit | 0.19 | 0.23 | 0.24 | 0.20 | 0.23 | 0.24 | 0.24 |
| Biodiversity | 0.24 | 0.26 | 0.24 | 0.23 | 0.23 | 0.22 | 0.21 |

*Simulating land cover based on results of the SMART analysis*: We assumed that while each landowner was rational and would choose the proportion of each land cover that provided the highest overall utility, he/she had imperfect knowledge of the value of their landscape, so the land cover choice was still probabilistic. For this prototype, we represented this probability simply by dividing the total utility of a single land cover choice by the sum of utilities over all three choices. Since the higher utility of one land cover choice was relative to the other choices, it would have a higher probability of being selected by the landowner. We then used the resulting probabilities to generate landscapes to use as inputs for breeding, migrating and wintering stages of a spatially explicit annual cycle model of grassland bird populations. For this prototype, we generated landscapes for each landowner type (three total) under each policy scenario (seven total), yielding 21 owner-policy combinations of simulations. Using this framework, we evaluated each of the 21 landowner-by-alternative combinations for its effect on grassland bird populations based on the land cover generated.

## References:

1. Evans TP, Kelley H. Multi-scale analysis of a household level agent-based model of landcover change. J Environ Manage. 2004; 72: 57-72.

2. Manson SM, Evans T. Agent-based modeling of deforestation in southern Yucatán, Mexico, and reforestation in the Midwest United States. Proc Natl Acad Sci USA 2007; 104: 20678-20683.

3. Edwards W. How to use multi-attribute utility measurement for social decision making. IEEE

Transactions on System, Man, and Cybernetics 1977; SMC-7: 326–340.
