# Supplementary material for: Strategic Grassland Bird Conservation throughout the Annual Cycle: Linking Policy Alternatives, Landowner Decisions, and Biological Population Outcomes
Source: PLoS One. 2015 Nov 16;10(11):e0142525. doi: 10.1371/journal.pone.0142525 (PMC4646652; doi:10.1371/journal.pone.0142525)
Supplement: S2 Appendix — Model details describe reproduction, carrying capacity, survivorship during spring and fall migration, winter survivorship, model simulation details and population summaries. (DOCX) [file pone.0142525.s002.docx]

# S2 Appendix: Intrinsic Grassland Bird Population Model

We developed a prototype spatially explicit annual cycle population model for grassland birds. Like many birds, the majority of grassland birds have four phases of their annual cycle that roughly correspond to the four seasons but occur in different locations, including: a summer breeding season in the north, a wintering period in the south and two migratory periods in the spring and fall when they travel between wintering and breeding areas. Thus, we assumed that reproduction occurs in one season and location and the majority of mortality occurs during the other three seasons. To reflect this basic biology, we modeled the population of birds in the breeding area at the start of the breeding season during year t (N_t_) as:

$N\text{t}=N\text{t-1}\left( 1+R\text{b} \right)S\text{f }S\text{w }S\text{s}$

where *R_b_* was the reproductive output during the summer breeding season and *S_f_, S_w,_* and *S_s_* were the survival probabilities during fall migration, over-winter, and spring migration, respectively. Rates were age-independent. We defined the values of each of these steps as a function of a landscape that consists of forest, grassland and agricultural patches. Results were summarized for a 30-year time period. We extended the model to reflect multiple stopover sites by adding stages to the fall and spring migration. If there were M stopover sites, then the final survivorship estimates for fall and spring migrations were simply the product of survivorship across M sites, such that:

$S\text{f}= \prod_{m=1}^{M} S\text{f,m}$

$S\text{s}= \prod_{m=1}^{M} S\text{s,m}$

Carrying Capacity:

Carrying capacity limits were built directly into our spatial model. The amount of habitat in each of the annual cycle phases/landscapes determined the number of birds that could survive and reproduce. We used a 30-meter pixel, a common resolution of remotely-sensed data, as the smallest spatial unit in the model. We assumed that up to 40 birds could persist on each 30-meter pixel in the wintering and stopover sites; the nest density and number of nest sites determined the carrying capacity in the breeding grounds and is defined below.

Reproduction (R_b_):

We used the group’s knowledge of grassland birds to determine how reproduction may change with respect to the composition and configuration of the landscape and used this information to parameterize the model described below. One important assumption was that grassland bird density and productivity were higher in larger grasslands than smaller ones (i.e., grassland birds are area-sensitive [1, 2]), although patterns of area sensitivity are known to vary regionally [3]. We assumed that grassland bird nest density and productivity were lower near edges, specifically within 25 meters of an edge [4, 5]. We also assumed that grassland bird abundance in a patch increased as the landscape around a patch became more grass-dominated [6, 7]. Building on these science-based assumptions, we developed a raster-based model to calculate the expected number of birds that would fledge from each pixel given the land cover of that particular pixel and the landscape context of surrounding pixels.

We determined the number of birds fledged by calculating the potential for nests to occur within a particular pixel, the survivorship probability of an individual from within an egg to independence, and the expected density of nests on the pixel. To determine nest potential, we assumed that only grassland pixels could support a nest (smallest, middle pixel; S2 Fig.). While grassland-associated predators are an important source of nest mortality [8], for this prototype model, we assumed constant “background” mortality from grassland-associated predators and focused on the edge effect of predation by woodland-associated animals. We assumed that predators were largely influenced by the amount of forest within 30 meters of the nest site. Translated to our raster model, as the number of pixels of forest within a one-pixel radius of the pixel where the nest was located increased, the survivorship of each egg decreased (medium-sized box in Fig. B1). To reflect patch size and the effect of the landscape on bird density (and subsequent nest density in the raster model), we varied potential nest density within a single grassland pixel depending on the surrounding land cover type. We assumed that forest or agriculture around a particular grassland pixel concentrated grassland bird nesting activity in that grassland pixel, resulting in higher potential nest densities compared to a grassland pixel surrounded by more grassland (S2 Fig. 1, S2 Table 1). Therefore, as the amount of grassland in the surrounding landscape increased, the density of nests within a pixel declined but the fledgling rate increased due to reduced edge effects.

**S2 Figure 1.** **An example landscape used for our rapid prototype model analyses.** Each 1.44-km^2^ landscape represented a hypothetical farm managed by an individual landowner. The three habitat classes are grassland (yellow), forest (green), and agriculture (brown). Nest density and survivorship were calculated for each pixel, based on the surrounding context (a 3 x 3 box around each pixel). Our modeling approach then incorporated the mean nest density of each total farm landscape as part of the bird population model.


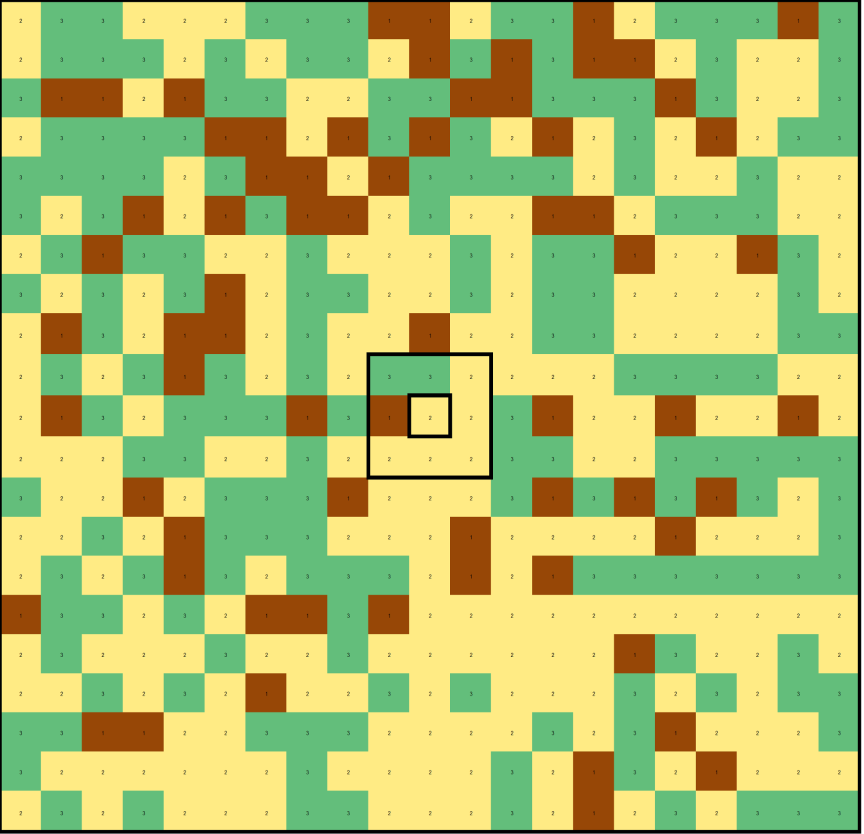


**S2 Table 1.** N**est density in grassland pixel as a function of land cover**. Trends were based on expert opinion. Species-specific empirical models could be directly incorporated in the future to enhance model accuracy. We assumed that forest or agriculture around a particular grassland pixel concentrated grassland bird nesting activity in that grassland pixel, resulting in higher potential nest densities compared to a grassland pixel surrounded by more grassland.

| **Surrounding pixel type** | **Nest density in grassland** |
| --- | --- |
| Agriculture | 1 |
| Grassland | 0.5 |
| Forest | 2 |

Survivorship during fall and spring migration (S_f_ and S_s_):

Compared to the breeding and wintering phases, much less is known about migratory stopover habitat use and survivorship phases, thus forcing us to rely exclusively on expert opinion solicited through consensus discussion among participants. Successful settlement of a site consists of two phases: detection of a suitable site and then, conditional on detection, settlement. We assumed that birds may not locate small or isolated high quality grassland sites within larger stands of forest. Once they settle at a site, they may forage in the surrounding landscape, and their survivorship probability increases with increasing forage quality of the surrounding landscape. We reflected this basic representation of stopover ecology by dividing stopover survivorship of an individual pixel into two components, occupancy and survivorship. Like reproduction, each component depended on the landscape composition at different scales.

Occupancy, the joint probability that a pixel is both located and utilized by migratory birds, was determined by the land cover type of the individual pixel and by the immediate surroundings (a 1-pixel neighborhood). At the pixel scale, we assumed that agriculture and grassland were suitable for a stopover but forest was not. To represent the effect of isolation on occupancy, the occupancy rate of a suitable pixel was determined by the amount of forest within 30 meters (one-pixel radius). As the amount of forest increased, occupancy rates declined [9].

Survivorship was determined by the forage quality within a one hectare “patch”, so we used a three-pixel by three-pixel neighborhood to evaluate forage quality. Both agriculture and grassland pixels provided forage. However, since grassland was assumed to provide higher quality forage it received a proportionally higher survivorship value.

Winter survivorship (S_w_):

We represented a grassland bird’s interaction with the landscape during winter in a similar way to the migration model. However, it was simplified in that only individual pixel-level determined survivorship (i.e., no neighborhood effect). We assumed that grassland birds did not use forest sites during the winter and that grassland pixels had higher survivorship than agricultural pixels. We further assumed that the overwinter survivorship rate was simply the average survivorship value of all suitable pixels, such that:

$S\text{w}= \frac{p\text{A }\omega\text{A }+ p\text{G} \omega\text{G}}{p\text{A }+ p\text{G}}$

where *p_A_* and *p_G_* were the proportion of agriculture and grassland in the wintering grounds, respectively, and $\omega\text{A}$ and $\omega\text{G}$ were winter survivorship rates of birds in agriculture and grassland pixels, respectively.

Grassland Bird Model Simulations and Population Estimates:

We initiated each simulation with a population of 100 birds and ran the model for 30 years. The eventual landowner decisions, to maintain or change land cover types within his/her landscape, were modeled on a per-pixel basis. We calculated the grassland bird population growth rate for each year and used average growth rate across 30 years of 50 iterations to evaluate the effectiveness of each policy alternative for grassland birds.

Due to time constraints, sensitivity analyses were not conducted to assess the impacts of uncertainty throughout the integrated modeling sequence, though such analyses will be important to develop these models further in the future. Additionally, exploring the impacts of combining multiple alternatives could be very informative.

## References:

1. Herkert JR, Sample DW, Warner RE. Management of midwestern grassland landscapes for the conservation of migratory birds. In: Thompson III, FR, editor. GTR-NC-187: management of midwestern landscapes for the conservation of neotropical migratory birds. St. Paul: USDA Forest Service, North Central Forest Experiment Station; 1996. pp. 89-116.

2. Ribic CA, Koford RR, Herkert JR, Johnson DH, Niemuth ND, Naugle DE, Bakker KK, Sample DW, Renfrew RB. Area sensitivity in North American grassland birds: patterns and processes. Auk 2009; 126: 233-244.

3. Winter M, Johnson DH, Shaffer JA, Donovan TM, Svedarsky WD. Patch size and landscape effects on density and nesting success of grassland birds. J Wildl Manage. 2006; 70: 158-172.

4. Johnson DH, Igl LD. Area requirements of grassland birds: A regional perspective. Auk 2001; 118: 24-34.

5. Renfrew RB, Ribic CA, Nack JL. Edge avoidance by nesting grassland birds: a futile strategy in a fragmented landscape. Auk 2005; 122: 618-636.

6. Ribic CA, Sample DW. Associations of grassland birds with landscape factors in southern Wisconsin. Am Midl Nat. 2001; 146: 105-121.

7. Renfrew RB, Ribic CA. Multi-scale models of grassland passerine abundance in a fragmented system in Wisconsin. Landsc Ecol. 2008; 23: 181-193.

8. Pietz PJ, Granfors DA, Ribic CA. Knowledge gained from video-monitoring grassland passerine nests. Studies in Avian Biology 2012; 43: 3-22.

9. Robertson BA, Doran PJ, Loomis ER, Robertson JR, Schemske DW. Avian use of perennial biomass feedstocks as post-breeding and migratory stopover habitat. PLoS ONE 2011; 6: e16941.
